# The Bottom-Line Mentality of Leaders in Education and Training Institutions: Where to Go for Innovation?

**DOI:** 10.3389/fpsyg.2021.689840

**Published:** 2021-07-02

**Authors:** Wenhai Wan, Longjun Liu, Jing Long, Qing Fan, Yenchun Jim Wu

**Affiliations:** ^1^School of Business Administration, Huaqiao University, Quanzhou, China; ^2^School of Business, Nanjing University, Nanjing, China; ^3^School of Management, Shanghai University, Shanghai, China; ^4^Graduate Institute of Global Business and Strategy, National Taiwan Normal University, Taipei, Taiwan; ^5^Leisure & Recreation Administration Department, Ming Chuan University, Taipei, Taiwan

**Keywords:** bottom-line mentality, relative deprivation, psychological safety, innovative behavior, person–organization values fit

## Abstract

According to the social exchange theory, this study analyzed how a bottom-line mentality (BLM) among leaders affects teachers' innovative behavior and how this relationship is mediated by relative deprivation and psychological safety and moderated by person-organization values fit. Using two stages of data collection, 491 responses from teachers were obtained and analyzed. The results revealed that leader BLM significantly negatively affected teachers' innovative behavior, and relative deprivation and psychological safety both partially mediated this influence of leader BLM. Person-organization values fit negatively moderated the positive effect of leader BLM on teachers' relative deprivation and the negative effect of leader BLM on teachers' psychological safety. This study enriches the current literature about BLM and tests the influence of leader BLM on teacher's innovation in the Chinese education and training institutions, and provides insights into favorable educational management practices.

## Introduction

Education is a process of teaching others to think with knowledge as a tool, thinking about how to create higher social wealth and realize the embodiment of self-worth. In the global competitive environment, how to practice good education is very important to cultivate innovative talents. Based on the restriction of public financial investment, the main direction of public education is to provide more and better basic education public services, while private education can provide more differentiated education services with high quality to meet people's individualized education needs. Only when public education and private education complement each other and cooperate with each other can we resolve the main contradictions in the field of education and better meet people's growing educational needs. Whether it is education and training institutions, or the operation of any category, market models and laws are changing with the development of the times and the progress of science and technology. Thus, innovation is undoubtedly the most important thing. Also, in the highly competitive education industry, the educational innovation practice and reform of the education and training institutions undoubtedly become important ways to obtain sustained competitive advantages (Senior, 2016; Curzi et al., 2019; Doménech-Betoret et al., 2019; Fernández-García et al., 2019). With the rapid development of modern educational technology and educational information, many fruitful achievements have been made in Chinese educational undertakings. According to the analysis report of Chinese education and training industry market prospect and investment strategy, the market size of Chinese education and training industry has reached 2.68 trillion RMB in 2018 and exceeded three trillion RMB in 2020. Teachers are key participants in education innovation, and how their innovative behavior can be fostered and the teachers motivated to innovate is a current direction of research. What's more, innovative behavior is a popular topic for scholars researching human resources management and organizational behavior, and most studies on this subject have been published in high-level journals. This paper focuses on the innovative behavior of teachers or trainers in education and training institutions. Generally speaking, education and training institutions refer to organizations that aim at academic education or adult continuing education, need the requirements of venues and teachers, need certification from the education authorities and obtain the qualification of running schools by social forces. Education and training institution is a kind of organization that gradually rises in recent years and makes knowledge and education resources information-based. This kind of institution contains educational information from pre-school education to universities, even doctors or going abroad, and also includes skills training for current workers or laid-off workers. It is a specialized institution whose main content is to provide educational resources and training information. The teaching content provided by education emphasizes the cultivation of students' mastery and application of basic theoretical knowledge. Training emphasizes the mastery and application of knowledge and skills closely related to enterprise work. Although the emphasis of education and training is different, it is undeniable that the innovative behavior of teachers and trainers in institutions is equally important.

In education, only when the top management give teachers support can creative processes be established and constructive proposals be formulated. However, because of the constant change in the management context, the leadership of an education and training organization is usually focused on a certain area of the organization that they consider the most critical while minimizing the attention given to other aspects (Wolfe, 1988). Management thus tends to reject creative processes and constructive ideas from teachers, which results in lower willingness of teachers to innovate. This mindset at the leadership level is referred to as the *bottom-line mentality (BLM)*. According to the research theme, we define leader BLM as the stubborn thinking that the administrators of educational and training institutions only pay attention to organizational interests and personal affairs, while ignoring the professional development of teachers and trainers in the institutions. In corporate, the obsession of management with bottom-line goals has led to various corporate scandals around the world. It's worth noting that in Chinese education industry, similar education scandals also exist and continue to occur. How to maintain the advancement and purity of Chinese education industry is a major issue to be considered by academia and education industry. Therefore, research on the BLM of leaders in Chinese education context can provide theoretical and practical implications to education practice in China. Studies have demonstrated that leader BLM has a certain negative effect on individuals, such as causing unethical (Mesdaghinia et al., 2019) and socially undermining (Greenbaum et al., 2012) behavior; however, whether BLM affects teachers' innovative behavior has not been reported. Therefore, ***how and when*** leader BLM affects teachers' innovative behavior is the topic we are concerned about.

In addition to organizational environment and their individual factors, leadership style and behavior are critical factors influencing innovative teacher behavior (Senior et al., 2014; Li et al., 2017; Cai et al., 2018; Naqshbandi et al., 2019; Rangus and Cerne, 2019). Studies discussing the effect of leadership factors on innovative behavior have predominantly focused on positive leadership styles or behavior (Javed et al., 2017; Schuckert et al., 2018; Zhang et al., 2018; Zhou and Wu, 2018; Fang et al., 2019), with few addressing the effect of leaders' thinking on innovative behavior. Moreover, most studies have investigated the positive effects of the environment, individual, and leadership factors on innovative behavior; those investigating the negative influences are few. The existing studies have made great contributions to the exploration of the influencing factors and motivations of innovative behavior; however, only limited dimensions of the topic have been revealed. In a dynamic educational environment, activating the positive factors and mitigating the negative factors affecting individual innovation is conducive to the sustainable development of the education organization (Papa et al., 2018). Accordingly, theoretical research on innovative behavior should not only consider the positive factors but also explore the elements that inhibit such behavior.

According to the theory of social exchange, people are always seeking the balance of resource exchange with others. When teachers feel that they are valued by the organization, they will have a sense of obligation to repay the organization, and they are more willing to carry out the innovative practices. On the contrary, the organizational environment that violates the principle of people-oriented management often makes teachers lose confidence in the organization, thus reducing their willingness to innovate. Leaders with high-level BLM show great importance to certain indicators, and at the same time, they will give hints of “only the bottom-line results,” and at the same time, they will ensure the realization of their bottom-line results through different rewards and punishments to teachers (Greenbaum et al., 2012). The leader BLM is more concerned with the profit of the organization, but in the eyes of teachers, the leader with BLM is more like abusing his power for the selfish desires. Therefore, this may improve teachers' sense of relative deprivation and reduce their psychological security, and ultimately harm teachers' innovative behavior. The theory of psychodynamics holds that psychological traits are the bridge connecting incentives and individual behaviors (Woodworth, 1918). Therefore, the present study inferred that leader BLM and bottom-line behaviors affect the psychological state of teachers, in turn influencing their innovative behavior. Based on social exchange theory, this study examined the inferred influence mechanism by using relative deprivation and psychological safety as factors connecting the cause and consequence. Scholars have not reached an agreement regarding the influence of relative deprivation on individuals; for example, Zoogah (2010) regarded such an influence to be positive, whereas Cole (2012) reported it to be negative. Moreover, because personal values dominate thinking and behaviors (Hemingway and Maclagan, 2004), whether the inferred influence mechanism is affected by the extent to which individuals' values match those of their organization requires investigation. Accordingly, person-organization values fit (P-OVF) was incorporated as a moderator in the influence mechanism.

Our research contributes to the existing literature in three major ways. First, it expands the research of BLM in the context of Chinese education management. Secondly, the bottom-line mentality scale is verified locally, which provides a reliable basis for subsequent local research. Thirdly, the specific mechanism and boundary conditions of the bottom-line mentality on teachers' innovative behavior are clarified. Of course, our research conclusions can inspire stimulating innovation vitality of education and training institutions in China and even around the world.

## Literature Review and Hypothesis

### Literature Review

The most commonly used definition of BLM is that proposed by Wolfe (1988), in which BLM is thinking in which leaders ignore competing for priorities to ensure bottom-line results (Greenbaum et al., 2012; Bonner et al., 2017; Mesdaghinia et al., 2019). The earliest studies on BLM predominantly emphasized its relationship with organizational performance; in particular, Eichenwald (2005) observed that ignoring ethical constraints to ensure certain bottom-line results was commonly considered beneficial to organizational profitability. However, recent studies on organizational behavior and human resources management have begun to systematically examine the potential defects in business leader BLM (Sims and Brinkman, 2002; Eichenwald, 2005; Greenbaum et al., 2012; Bonner et al., 2017; Mawritz et al., 2017).

In most qualitative studies, scholars have associated BLM in top management with negative results; for example, leader BLM has been revealed to result in unethical employee and organization behaviors or to create a negative organizational environment; these negative results ultimately lead to business closedown (Sims and Brinkman, 2002; Mandis, 2013). Quantitative studies on the organizational level have revealed various negative influences of leader BLM on organizations and individuals. For example, Greenbaum et al. (2012) discovered that BLM did not exist only among leaders; such BLM was passed on to their subordinates, who subsequently had negative influences on their colleagues, organizations, and society. Mawritz et al. (2017) observed that leaders with BLM prioritize corporate profitability and self-interest, and when leaders with high BLM are confronted with subordinates who have overstepped their authority, the leaders have impaired self-regulation (i.e., lost their self-control) and become abusive toward the subordinates. Based on the aforementioned studies, Mesdaghinia et al. (2019) proposed that leader BLM was profit-oriented, induced unethical leader behavior in subordinates, and ultimately increased the desire of subordinates with high ethical standards to resign. The latest research extends the influence of leader BLM to constructive outcomes for individuals and organizations, which is a breakthrough. For example, the literature explores how leader BLM undermines or promotes performance and team creativity. Babalola et al. (2020) draw on the social exchange theory and point out that BLM of senior managers can affect employees' sense of responsibility to the bottom line, thus affecting employees' task performance and unethical pro-organizational behavior. However, Quade et al. (2020) point out that supervisors with BLM will have a harmful social exchange relationship with employees and have a negative impact on employees' task performance, which will actually have a negative impact on the organization's bottom line. Also, Greenbaum et al. (2020) believe that high BLM protocols in teams enhance the target shielding effect of BLM teams, which will have more serious consequences on team psychological security and thus reduce team creativity.

According to these aforementioned studies, the various consequences of leader BLM (e.g., unethical behavior, a tendency to resign among subordinates, and abusive supervision) have been a focus in recent studies. Although both qualitative and quantitative studies have revealed the negative effects of BLM on organizations and individuals, few studies have discussed the influence of leader BLM on individual constructive behavior, and most studies have been conducted from a single perspective (i.e., ethics). Also, BLM is a worldwide phenomenon, but studies on leader BLM in the context of Chinese management are few, which has been highlighted by scholars promoting leadership localization (Chen and Fahr, 2001). It is noteworthy that BLM may also exist among leaders of the education industry. BLM is the research frontier, but it has not been studied in the education industry. Accordingly, the present study, using social exchange theory as its basis, determined the influence of leader BLM on innovative teacher behavior to fill the gap in the literature on leader BLM; additionally, the consequences of leader BLM in the context of China's education management was examined.

### Leader BLM and Teacher Innovation Behavior

Social exchange theory has been widely used in sociology (Blau, 1964), psychology, and other disciplines in the last century. It is among the most commonly used theoretical frameworks for explaining individual behavior in the workplace. Social exchange relationships in the workplace have gradually attracted the attention of research institutes in the field of management, such as exchanges between leaders and members, which describe exchange relationships between higher and lower levels; organizational support, which describes exchange relationships between individuals and organizations; and exchange relationships between employee groups and teams. Blau (1964) suggested that social exchange is different from economic exchange. Social exchange involves a wider range of resources and information, and the exchange relationship in the organization is based on individual voluntary behaviors generated by the behaviors of others or other organizations within the platform organization. Social exchange relationships follow a certain moral standard and that equality and reciprocity are the basic principles of exchange relationships. When this balance mechanism is broken, the behavioral responses of both parties tilt or even cease. Social exchange theory has been widely used in studies on CSR, employee behavior, and performance output.

According to the reviewed literature, leader BLM is the state in which leaders focus on areas of the business that they prioritize and ignore the importance of other aspects to ensure the realization of bottom-line goals. Such one-dimensional thinking reveals a leader's values as opposed to their leadership style; leader BLM is usually latent and has been verified to exert strong influences on individuals' behaviors. According to the academia concept of innovation behavior, teacher innovation behavior is the process through which teachers put their thinking into practice and implement their creativity within their organization to solve a difficult situation they are in (Janssen et al., 2010; Zhu and Zhang, 2019). For education in a competitive environment, innovative teacher behavior is critical to sustaining a competitive advantage and achieve sustainable development for schools.

Extreme manager thinking and behaviors are imitated by subordinates (Bono and Ilies, 2006) and manifest particularly in individuals' exchange relationships with their leader and organization. The present study considered leader BLM, one type of extreme thinking, to be a hindrance to individuals' innovative behavior. Studies on leadership have demonstrated that leaders are a symbol of power and status and also a crucial source of information for subordinates. A leader with BLM prioritizes organization profits or self-interest (Wolfe, 1988) and shows little concern for or even disregards subordinates' career development, resulting in these subordinates having weak insider identity and a failure to provide material and spiritual incentives to highly performing subordinates. According to social exchange theory (Blau, 1964), when a leader has BLM, teachers—particularly those in education front and scientific research—have lower creative input, shorten the process of innovation when addressing internal organizational goals and research and development tasks, and even exhibit anti-innovation deviant work behavior. In schools, although leader BLM is associated with high profits (Wolfe, 1988), leaders usually ignore some values such as internal social responsibility, the ethical bottom-line, and teacher needs during the process of achieving bottom-line goals; this often exerts destructive effects on teachers and organizations. Moreover, BLM, a passive mindset of leaders (Keeler and Webster, 2018), can result in leaders deviating from human-centered management and ignoring the overall school goals. Thau and Mitchell (2010) suggested that passive leader behaviors are crucial antecedents of ego depletion and negatively affect individuals' self-efficacy. Finally, the innovative behaviors that individuals can exhibit have evolved. Teachers now require more resources and support from their colleagues if they are to demonstrate their innovation ability and formulate innovative education proposals (Premaratne, 2001). Leaders with BLM do not emphasize teachers' self-fulfillment, care only for profits and their self-interest, and tend to be conservative and traditional (Wolfe, 1988); teachers are thus less likely to obtain behavioral support and the required resources for innovation from such leaders. Accordingly, this study proposed the following hypothesis:

H1: Leader BLM significantly negatively affects teacher innovation behavior.

### Mediating Effects of Relative Deprivation and Psychological Safety

Psychodynamics holds that the psychological traits of an individual connect their incentives and behaviors (Woodworth, 1918). Therefore, this study inferred that the relative deprivation and psychological safety of teachers are crucial factors connecting leader BLM and teacher innovation behavior. The concept of relative deprivation was introduced by Stouffer et al. (1949). According to the operational definition of relative deprivation proposed by Runciman (1966) concerning to its formulation, an individual feels relatively deprived of X only when the following four criteria are met: the individual does not have X, realizes that others have X, desires to have X, and considers such a desire feasible. Relative deprivation is a consequence of unfairness and a subjective perception (Walker, 1999). When an individual feels deprived of their basic rights, this sense of deprivation damages the individual's psychological development. Within the framework of this study, we define the sense of relative deprivation as the perceived unfair treatment of teachers and trainers, which mainly comes from leaders with BLM.

Subordinates' relative deprivation, a psychological trait, is a key factor connecting the leader BLM with innovative subordinates' behavior (Zhang and Zhang, 2016). The psychological cognition of organization members is strongly influenced by leaders' behaviors and traits. Leaders with BLM tend to ignore priorities; specifically, all things are considered unimportant compared with school profits and the leader's self-interest, which is unfair to teachers. According to social exchange theory (Blau, 1964), people maximize their self-interest during interactions with others. Therefore, when their leader has BLM, subordinates perceive procedural injustice and experience relative deprivation. In schools, individual teachers' sense of self-fulfillment is dependent on leaders' support and implementation of their innovative proposals; a mismatch between the leader's attitudes and behaviors and the subordinate's expectations leads to the subordinates perceiving the situation negatively (Leineweber et al., 2014). Furthermore, leaders have authority and must be reliable for subordinates (Treviño et al., 2014). Accordingly, subordinates scrutinize their leader's thinking and mentality during interactions with them; this scrutiny is critical to the establishment of a trusting relationship between the two parties. Leader BLM results in a weaker trusting relationship, and weaker trust adversely affects subordinates' mental abilities (Pierce and Gardner, 2004). Therefore, when a teacher's creative thinking is not trusted and recognized but that of others is being supported, the teacher considers their creation to have been rejected and experiences increased relative deprivation. Leaders with strong BLM are obsessed with their success and survival in the competitive environment and thus ignore the needs and demands of people surrounding them (Bonner et al., 2017), which results in subordinates having the mindset that they will be rewarded solely for their contribution to their leader rather than that to their organization (Mesdaghinia et al., 2019). Such a mindset is inarguably a blow to teachers who dedicate themselves to their organizations and improving educational performance, because such teachers are potentially abused when they fail to satisfy the demand of their leader with high BLM, which in turn intensifies their perception of unfairness and hence their relative deprivation.

Moreover, this study posited that teachers' relative deprivation inhibits their innovative behavior. The proactiveness and individual capacity of a teacher are not only impeded by the lack of material incentive but also by the teacher's relative deprivation. When teachers perceive unfair treatment, their relative deprivation increases, and organizational commitment decreases, reducing the extent of their innovative behavior (Zigarmi et al., 2009). According to social exchange theory (Blau, 1964), unfair procedures lead to counterproductive subordinates' work behavior, namely withdrawal (Smith et al., 2012). In schools, teacher withdrawal behavior is a major cause of lack of innovation. When subordinates experience relative deprivation, they tend to respond negatively (Smith et al., 2012). Negative teacher behavior predominantly manifests as anti-innovation behavior in school. Accordingly, the present study proposed the following hypothesis:

H2a: Relative deprivation mediates the relationship between leader BLM and teacher innovation behavior.

Psychological safety refers to the belief of individuals that they can express their genuine thoughts and viewpoints at work without having to worry about the repercussions of their speech and behavior (Edmondson, 1999; Wang et al., 2018; Plomp et al., 2019). In the context of educational organization, we define the psychological safety of teachers and trainers as their psychological perception of the surrounding environment (from colleagues, leaders, physical environment, etc.) when they complete organizational affairs and innovative work. According to the reviewed literature, leader BLM results in greater relative deprivation of subordinates and affects subordinates' psychological safety, which is an individual psychological trait (Nembhard and Edmondson, 2006). Based on the definition of BLM, a leader narrowing their attention to personal matters, self-interest, or a specific area of business hinders sustainable business development and subordinates' individual development (Wolfe, 1988). Furthermore, social exchange theory (Blau, 1964) holds that human relationships are essentially a type of social exchange relationship; therefore, when leaders with BLM exhibit a profit-oriented mindset or treat the subordinates unfairly, the subordinate experiences psychological insecurity. Based on H2a, leader BLM destroys the trust between leaders and teachers. Teachers' mental abilities are diminished when their trust in their leader is low, and their psychological safety is poor when the leader's behaviors and attitudes do not align with the teachers' expectations. Finally, in schools, negative emotional signals are generated when teachers propose innovative ideas to a leader with BLM, and the leader's rejection of constructive innovative ideas leads to psychological insecurity among the teachers.

Psychological safety is a pre-requisite to subordinate innovation (Hood et al., 2016) and a crucial driver of individuals' willingness to internalize certain roles. Based on the social exchange theory of Blau (1964), teachers with high psychological safety are more able to perceive colleagues' support for innovative activities and are thus more willing to innovate (Moore and Wang, 2017); innovative activities gradually and naturally succeed as the innovative identity of teachers is strengthened. Studies have indicated that psychological safety critically links incentives and individual capacity (Walker, 1999; Hu et al., 2018) and affects individuals' intrinsic motivation (Kahn, 1990; Chen et al., 2019). Similar to how external stimuli elicit individual innovative behavior through intrinsic motivation, external factors (e.g., leaders' behavior, organizational atmosphere, and job characteristics) affect innovative teacher behavior through intrinsic motivation (Shalley et al., 2009). Accordingly, the present study proposed the following hypothesis:

H2b: Psychological safety mediates the relationship between leader BLM and teacher innovation behavior.

### Moderation of P-OVF

P-OVF refers to the level of fit between individual and organizational values (Schwartz, 1992). Good fit indicates consistency between an individual's values and those of their organization. Regardless of the organization or individual, values serve as the beliefs that will prevail in all contexts, and these beliefs strongly influence the capacity, psychological perceptions, and decision-making of individuals (Valentine et al., 2002). Individuals for whom the P-OVF is good are highly loyal (Kristof, 1996), and high loyalty results in a high tolerance for the leader or organization, which in turn weakens the teachers' perceptions of unfairness. Therefore, such teachers tend to analyze problems from their perspective and find ways to solve these problems as opposed to feeling treated unfairly by their leader or organization and thus experience less relative deprivation. By contrast, teachers for whom the P-OVF is poor consider themselves a misfit in their organization and thus have a weak sense of belonging to their organization and exhibit less organizational citizenship behavior (Edwards and Cable, 2009). When this type of teacher works with leaders with BLM, they tend to analyze problems from the perspective of external factors, are prone to feelings of being abused by their leader, and therefore easily experience relative deprivation.

The present study posited that P-OVF moderates the mediating effect of relative deprivation in the relationship between leader BLM and innovative teacher behavior. Teachers with favorable P-OVF are tolerant and understanding of their organization and leader (Kristof, 1996) and hence do not usually experience relative deprivation when interacting with a leader with BLM. Moreover, teachers with favorable P-OVF often have a sense of ownership of their work and are motivated to contribute to their organization (Edwards and Cable, 2009), enabling them to use their creativity effectively. By contrast, teachers with poor P-OVF tend to have low trust in and a low sense of belonging toward their organization, which possibly leads to high relative deprivation and makes the teachers reluctant to innovate for their organization. Accordingly, the present study proposed the following hypothesis:

H3a: Teachers' P-OVF negatively moderates the relationship between leader BLM and teachers' relative deprivation. Specifically, favorable P-OVF results in a weak positive effect of leader BLM on teachers' relative deprivation and moderates the mediating effect of relative deprivation on the relationship between leader BLM and teacher innovation behavior.

Similarly, for teachers with a high matching between personal and organizational values, they can perceive that organizational values can satisfy personal psychological expectations of organizations. Therefore, even when faced with leaders with BLM, they have expectations for leadership and organization, and can strengthen their personal beliefs. This means that the uncertainty perceived by teachers in the organization is reduced, and teachers have a higher sense of psychological security, thus enhancing teachers' job satisfaction and improving their innovative motivation (Shafer, 2002). On the other hand, teachers with a low matching degree of personal-organizational values are more likely to generate stress in their work (Hartnell et al., 2011). Especially in the face of leaders with BLM, when they perceive that creative proposals cannot be achieved, they will have great pressure of self-realization, which can cause serious damage to teachers' psychology, and eventually lead to the decrease of teachers' psychological security in the organization.

According to the flexible orientation of organizational values-paying attention to the external corresponding vitality dimension, organizational values put more emphasis on exerting teachers' creativity and improving their flexibility and adaptability (Quinn and Rohrbaugh, 1983; Shafer, 2002). As mentioned above, when teachers' personal-organizational values match well, individuals' psychological security will increase when interacting with leaders with BLM, and teachers' commitment to the organization will increase accordingly, thus prompting teachers to be more willing to innovate. Therefore, the present study proposed the following hypothesis:

H3b: Teachers' P-OVF positively moderates the negatively relationship between leader BLM and teachers' psychological safety. Specifically, favorable P-OVF is associated with a weaker negative effect of leader BLM on teachers' psychological safety and moderates the mediating effect of psychological safety in the relationship between leader BLM and teacher innovation behavior.

**Figure 1:**
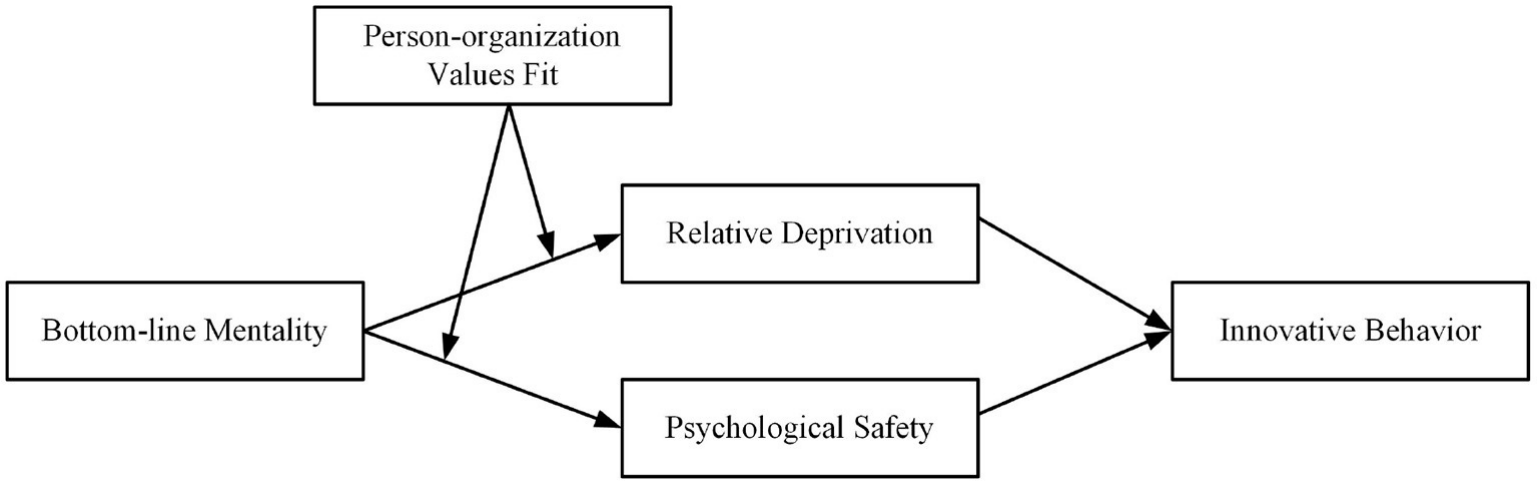
Theoretical model.

The research model in Figure 1.

## Methods

### Participants and Procedures

The research data were collected via email and online questionnaires from teachers in 37 education and training institutions located in Fujian, Guangdong, Jiangsu, Zhejiang, Shanghai, and Beijing in China. These teachers and trainers are front-line workers in the organization, and they have rich experience in teaching and training. However, we ensure that the participants of the two stages are paired, that is, the data of the two stages come from the same participant. Two stages of data collection were conducted. In the first data collection stage in early July 2020, participants completed a questionnaire comprising a leader BLM scale and P-OVF scale. The participants' responses were numbered to pair them with the corresponding responses in the next data collection. The methods to eliminate invalid samples were: the scores of 8 consecutive samples were consistent, the filling time was <1 min, or at least one item was ignored. In total, 650 questionnaires were distributed, with 621 returned and 577 were valid. In the second data collection stage, conducted at the beginning of late October 2020, participants completed a questionnaire composed of a relative deprivation scale, psychological safety scale, and innovation behavior scale; 577 questionnaires were distributed and 551 returned with 503 valid. Eliminating the invalid responses once again, this study obtained 491 valid responses. Regarding the teacher participants, 50.1% were men; 71.5% were aged younger than 40 years; 51.8% are master's and doctor's degrees; 72.1% had worked for more than 3 years.

### Measures

Scales verified in foreign studies were used, and a 5-point Likert scale was employed to rate all questionnaire items, with the scale ranging from 1 (*completely disagree*) to 5 (*completely agree*). Teachers of the English language and management were recruited to conduct back translation; subsequently, the translated scales were modified by two teachers of management and psychology with reference to the original scales before finalizing the Chinese version of the scales (Table 1).

**Table 1:** Items and test results of reliability and validity.

**Variables**	**Items**	**Loading**	**Cronbach's α**	***CR***	***AVE***
Bottom-line mentality	My leader is more concerned about profit than the happiness of teachers	0.804	0.754	0.846	0.581
	My leader only cares about realizing his bottom line	0.791			
	My leader only cares about his business	0.703			
	My leader regards the bottom line as more important than anything else	0.746			
Relative deprivation	I feel unfairly treated in the organization	0.790	0.726	0.846	0.648
	I feel that I am in a worse position than others in the organization	0.783			
	Compared with others, I am not satisfied with the situation I am facing now	0.839			
Psychological safety	Members of the organization I work in can raise questions and express their opinions freely	0.710	0.866	0.897	0.556
	If I make a mistake in the organization, other members will not have an opinion on me	0.772			
	Members of my organization do not exclude people who are different from them	0.773			
	My organization supports risk-taking behavior	0.744			
	In my organization, there is no difficulty in seeking help from others	0.732			
	In my organization, no one will deliberately undermine my efforts	0.733			
	In the organization, my skills and talents will be valued	0.755			
Innovative behavior	I often think of innovative ideas at work and education	0.814	0.848	0.898	0.689
	I will seek new ideas and ways to solve the problems faced by my work in teaching	0.843			
	I will have breakthrough ideas in related teaching fields	0.845			
	I will play a good demonstration role in creativity	0.817			
P-O values fit	My values are consistent with the culture in my organization	0.912	0.878	0.925	0.804
	I think my personal life values and organizational values are similar	0.886			
	The culture and values advocated by the organization are in line with my life values	0.893			

#### Bottom-Line Mentality (BLM)

The scale was adapted from Greenbaum et al. (2012), had a Cronbach's α of 0.754, and comprised four items. An example of an item is “My leader is more concerned about profit than the happiness of teachers.”

#### Relative Deprivation (RD)

The scale was adapted from Tropp and Wright (1999), had a Cronbach's α of 0.726, and was composed of three items. An example of an item is “I feel unfairly treated in the organization.”

#### Psychological Safety (PS)

The scale was adapted from Edmondson (1999), had a Cronbach's α of 0.866, and contained three forward-scored and four reverse-scored items. An example of a forward-scored item is “Members of the organization I work in can raise questions and express their opinions freely,” whereas an example of a reverse-scored item is “The organization I work in does not support adventurous behavior.”

#### Innovation Behavior (IB)

This scale was developed on the basis of Scott and Bruce (1994) and comprised four items adapted from relevant domestic studies. The scale had a Cronbach's α of 0.848, and an example of an item is “I often think of innovative ideas at work and education.”

#### Person-Organization Values Fit (P-OVF)

The scale was adapted from Cable and Derue (2002), had a Cronbach's α of 0.878, and contained three items. An example of an item is “My values are consistent with the culture in my organization.”

#### Control Variables

According the past studies, the individuals' relative deprivation, psychological safety, and innovative behavior may be affected by their sex, age and years of work experience (Obrenovic et al., 2020), so we incorporated these as control variables to obtain robust research results. The coding of the variables was as follows: for sex, 1 = men and 2 = women; for age, 1 = 24–30 years, 2 = 31–40 years, 3 = 41–50 years, and 4 = older than 50 years; and for education; 1 = undergraduate, 2 = master, and 3 = doctor; and for work experience, 1 = <3 years, 2 = 3–6 years, 3 = 6–9 years, and 4 = 10 years or more.

### Reliability and Validity Tests

This study used the internal consistency coefficient (Cronbach's α) and composite reliability (CR) to assess the reliability of each scale. Table 1 reveals that all scales have a Cronbach's α coefficient higher than 0.700 and a CR higher than 0.800, indicating that the proposed questionnaire has satisfactory reliability.

To examine the discriminant validity of the variables, this study used AMOS22.0 to conduct confirmatory factory analysis on BLM, P-OVF, relative deprivation, psychological safety, innovation behavior, and the competing models. The analysis results are presented in Table 2. The goodness of fit of the five-factor model (χ^2^ = 397.045, df = 179, χ^2^/df = 2.218, comparative fit index = 0.946, incremental fit index = 0.947, Tucker–Lewis index = 0.937, root-mean-square residual = 0.044, root-mean-square error of approximation = 0.050) is considerably higher than that of the other models, indicating high discriminant validity in the five factors (Hu and Bentler, 1999).

**Table 2:** Confirmatory factor analysis results.

**Models**	**χ^**2**^**	**df**	**χ^2^/df**	**CFI**	**IFI**	**TLI**	**RMR**	**RMSEA**
Five-factors model	397.045	179	2.218	0.946	0.947	0.937	0.044	0.050
Four-factors model	1202.161	183	6.569	0.750	0.751	0.713	0.104	0.107
Three-factors model	1513.362	186	8.135	0.674	0.676	0.632	0.123	0.121
Two-factors model	2093.706	188	11.135	0.532	0.534	0.477	0.137	0.144
Single-factor model	2417.989	189	12.794	0.452	0.391	0.455	0.142	0.155

*BLM, Bottom-line mentality; RD, Relative Deprivation; PS, Psychological Safety; IB, Innovative Behavior; P-OVF, P-O Values Fit; Five-factors mode: BLM, P-OVF, PS, RD, IB; Four-factors model: BLM+P-OVF, PS, RD, IB; Three-factors model: BLM+P-OVF, PS+RD, IB; Two-factors model: BLM+P-OVF, PS+RD+IB; Single-factor model: BLM+P-OVF+PS+RD+IB*.

### Common Method Bias Test

To prevent potential common method bias, this study employed a multisource, multistage data collection method. Additionally, the participants were informed that their responses would be kept confidential to ensure the authenticity and validity of the responses. Harman's single factor test (Chen and Lim, 2012) was employed to conduct factor analysis of the responses to all items of the five factors. In principal component analysis, five factors were extracted, and the first unrotated principal component accounted for 26.60% of the variance, which did not exceed the threshold of 40%. Therefore, the common bias was non-significant (Podsakoff et al., 2003).

## Results

### Correlation Analysis

Table 3 presents the means, standard deviations, and correlation coefficients of the variables. Leader BLM was discovered to be significantly negatively correlated with teacher's innovation behavior (*r* = −0.317, *p* < 0.001) and psychological safety (*r* = −0.240, *p* < 0.001), and significantly positively correlated with teachers' relative deprivation (*r* = 0.411, *p* < 0.001). Teachers' psychological safety and innovative behavior are significantly positively correlated (*r* = 0.388, *p* < 0.001). Teachers' relative deprivation and innovative behavior are significantly negatively correlated (*r* = −0.259, *p* < 0.001). These correlation results provided a basis for the subsequent analyses.

**Table 3:** Means, standard deviations and correlation coefficients of variables.

**Variables**	***M***	***SD***	**1**	**2**	**3**	**4**	**5**	**6**	**7**	**8**
1 Genda	–	–								
2 Year	2.06	0.90	−0.070							
3 Education	1.62	0.66	0.005	−0.007						
4 Work experience	2.40	0.94	−0.016	0.267^***^	−0.111^*^					
5 BLM	2.91	0.78	0.054	−0.011	0.167^**^	−0.060				
6 P-OVF	3.17	0.91	0.027	−0.009	0.094	−0.081	0.060			
7 PS	3.68	0.79	−0.005	0.037	0.000	0.028	−0.240^***^	0.085		
8 RD	2.86	0.82	0.065	0.058	0.036	−0.044	0.411^***^	−0.146^**^	−0.272^***^	
9 IB	3.53	0.80	0.033	−0.014	−0.011	0.041	−0.317^***^	0.137^**^	0.388^***^	−0.259^***^

*BLM, Bottom-line mentality; RD, Relative Deprivation; PS, Psychological Safety; IB, Innovative Behavior; P-OVF, P-O Values Fit;*

* *p < 0.05*,

** *p < 0.01*,

*** *p < 0.001*.

### Hypothesis Test

This study conducted regression analysis and concurrently tested the multicollinearity of the variables. Variance inflation factor analysis revealed that the variance inflation factor was lower than two for all variables, indicating no multicollinearity in the research model.

Regression analysis was conducted using SPSS22.0 with control of the demographic variables. Table 4 presents the regression coefficients between all variables and the summary of each model. The regression analysis for the main effect revealed that leader BLM has a significant negative effect on teacher's innovation behavior (β = −0.335, *p* < 0.001, M6); thus H1 was supported. Besides, we also found that leader BLM has a significant negative effect on teacher's psychological safety (β = −0.250, *p* < 0.001, M4), and has a significant positive effect on teacher's relative deprivation (β = 0.433, *p* < 0.001, M2). At last, as we can see in the Table 4, teacher's psychological safety has a significant positive effect on their innovation (β = 0.394, *p* < 0.001, M7), and the relative deprivation has a significant negative effect on their innovation (β = −0.256, *p* < 0.001, M8). The test of direct effects provide a basis for further analysis.

**Table 4:** Regression analysis results of direct and mediating effects.

	**RD**		**PS**		**IB**					
**Variables**	**M1**	**M2**	**M3**	**M4**	**M5**	**M6**	**M7**	**M8**	**M9**	**M10**
Genda	0.114	0.078	−0.004	0.017	0.052	0.079	0.053	0.081	0.074	0.091
Year	0.072	0.070	0.028	0.029	−0.021	−0.20	−0.032	−0.003	−0.030	−0.009
Education	0.036	−0.047	0.003	0.051	−0.008	0.057	−0.009	0.002	0.039	0.049
Work experience	−0.053	−0.037	0.017	0.008	0.040	0.028	0.033	0.026	0.025	0.022
BLM		0.433^***^		−0.250^***^		−0.335^***^			−0.251^***^	−0.269^**^
PS							0.394^***^		0.335^***^	
RD								−0.256^***^		−0.152^**^
*R^2^*	0.013	0.178	0.002	0.061	0.003	0.106	0.154	0.071	0.208	0.126
*ΔR^2^*	0.013	0.165	0.002	0.059	0.003	0.102	0.151	0.067	0.103	0.020
*ΔF*	1.577	97.137^***^	0.930	30.337^***^	0.408	55.570^***^	86.447^***^	35.056^***^	62.681^***^	10.961^**^

*BLM, Bottom-line mentality; RD, Relative Deprivation; PS, Psychological Safety; IB, Innovative Behavior; P-OVF, P-O Values Fit;*

** *p < 0.01*,

*** *p < 0.001*.

A method proposed by Baron and Kenny (1986) was used to test the mediating effects of teachers' relative deprivation and psychological safety. For the mediating effect of teachers' relative deprivation, BLM and relative deprivation were used as independent variables and innovative behavior as the dependent variable. Seen as Table 4, the regression result suggested a significant negative effect of BLM on teacher innovative behavior, but the regression coefficient is reduced (β = −0.269, *p* < 0.01, M10); relative deprivation has a significant negative effect on teacher innovative behavior (β = −0.152, *p* < 0.01, M10). Accordingly, relative deprivation has a partial mediating effect on the relationship between BLM and teacher innovative behavior, supporting H2a. To test the mediating effect of psychological safety, BLM and psychological safety were used as independent variables and innovative behavior as the dependent variable. The regression analysis revealed a significant negative effect of BLM on teacher innovation behavior, but the regression coefficient is reduced (β = −0.251, *p* < 0.001, M9); psychological safety exerts a significant positive effect on innovative behavior (β = 0.335, *p* < 0.001, M9). Accordingly, psychological safety exerts a partial mediating effect on the relationship between BLM and teacher innovation behavior, supporting H2b.

**Figure 2:**
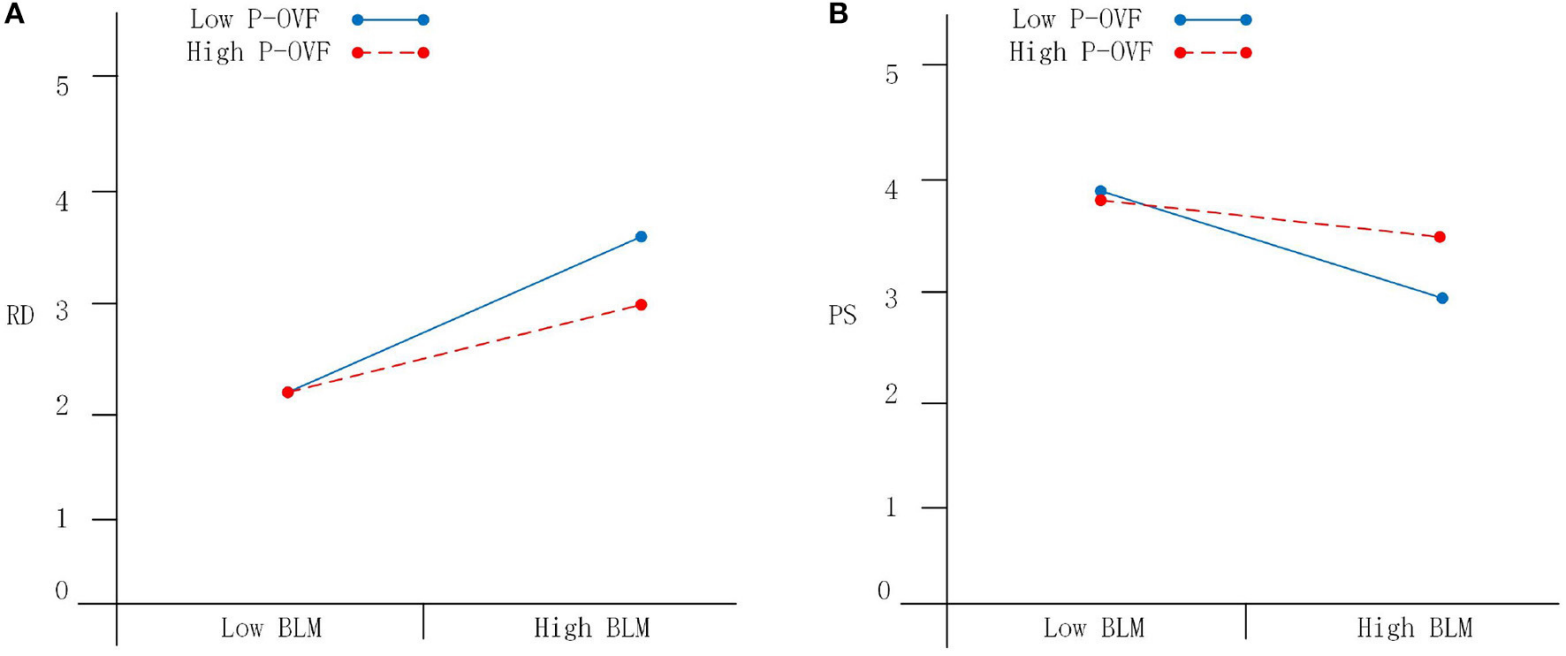
**(A, B)** The moderating effect of P-OVF on the relationship between BLM, RD, and PS. BLM, Bottom-line mentality; RD, Relative Deprivation; PS, Psychological Safety; P-OVF, P-O Values Fit.

Subsequently, the moderating effect of P-OVF was tested. Before the test, leader BLM and teachers' P-OVF were both centered, and their interaction term was obtained. The BLM, P-OVF, and interaction term were employed as independent variables and relative deprivation as the dependent variable in regression analysis. Seen as Table 5, The result revealed that the interaction term between BLM and P-OVF has a significant negative effect on relative deprivation (β = −0.126, *p* < 0.01, M3), indicating that P-OVF negatively moderates the relationship between BLM and relative deprivation, partially supporting H3a. The BLM, P-OVF, and interaction term were employed as the dependent variable and psychological safety as the independent variable in another regression analysis. The interaction term has a significant positive effect on psychological safety (β = 0.153, *p* < 0.01, M6), indicating that P-OVF negatively moderates the negatively relationship between BLM and psychological safety, partially supporting H3b (Table 5). Specific moderation effect are shown in Figures 2A,B.

**Table 5:** Regression analysis results of moderation effects.

	**RD**			**PS**		
**Variables**	**M1**	**M2**	**M3**	**M4**	**M5**	**M6**
Genda	0.114	0.085	0.079	−0.004	0.013	0.021
Year	0.072	0.073	0.065	0.028	0.028	0.038
Education	0.036	−0.030	−0.034	0.003	0.042	0.047
Work experience	−0.053	−0.048	−0.043	0.017	0.014	0.007
BLM		0.440^***^	0.433^***^		−0.254^***^	−0.245^***^
P-O Value Fit		−0.156^***^	−0.152^***^		0.086^*^	0.081^*^
BLM × P-O Value Fit			−0.126^**^			0.153^**^
*R^2^*	0.013	0.207	0.222	0.002	0.070	0.093
*ΔR^2^*	0.013	0.195	0.014	0.002	0.068	0.023
*ΔF*	1.577	59.379^***^	8.923^**^	0.216	17.781^***^	12.193^**^

*BLM, Bottom-line mentality; RD, Relative Deprivation; PS, Psychological Safety; IB, Innovative Behavior; P-OVF, P-O Values Fit;*

* *p < 0.05*,

** *p < 0.01*,

*** *p < 0.001*.

Finally, the PROCESS macro for SPSS was used in conjunction with the bootstrap procedure to test the moderated mediation (Hayes, 2013). Innovative behavior was the dependent variable, BLM the independent variable, P-OVF the moderator, and relative deprivation and psychological safety the mediators (Table 6). The bootstrap sample size was 5,000, and the 95% confidence interval (CI) was used. The analysis result revealed that the size of the moderated mediation effect of relative deprivation is 0.019 (standard error = 0.008, 95% CI = [0.006, 0.041]). This indicated a significant moderated mediation effect and thus supported H3a. The size of the moderated mediation effect of psychological safety is 0.051 (standard error = 0.015, 95% CI = [0.024, 0.084]), indicating a significant moderated mediation effect and thereby supporting H3b.

**Table 6:** Moderated mediation effect test of BOOTSTRAP method for PROCESS macro.

**Moderation Variable: P-OVF**	**Effect**	**SE**	**DCI**	**UCI**
	BLM → RD → IB		
High	−0.048	0.019	−0.097	−0.018
Low	−0.083	0.027	−0.140	−0.033
Moderated Mediation Effect	0.019	0.008	0.006	0.041
	BLM → PS → IB		
High	−0.035	0.022	−0.084	−0.004
Low	−0.129	0.030	−0.200	−0.077
Moderated Mediation Effect	0.051	0.015	0.024	0.084

*BLM, Bottom-line mentality; RD, Relative Deprivation; PS, Psychological Safety; IB, Innovative Behavior; P-OVF, P-O Values Fit*.

## Discussion

### Conclusions

Innovation is a crucial means with which the education and training industry achieve a competitive advantage and has thus received considerable research attention. To analyze the causal relationship between leader BLM and teacher innovation behavior, this study constructed a theoretical model comprising leader BLM, relative deprivation, psychological safety, and teacher innovative behavior and incorporated P-OVF as a moderating variable. The causal relationships, mediating effects, and moderating effects among these variables were analyzed. The obtained results supported the proposed hypothesis.

Leader BLM significantly negatively affects teacher innovation behavior. This result indicates that the prioritization of only education and training industry profit by leaders results in less innovative behaviors of teachers during work, tasks, and interactions with colleagues. Relative deprivation and psychological safety both mediate the relationship between leader BLM and teacher innovation behavior. Thus, leader BLM reduces the amount of teacher innovation behavior through its effect on teachers' psychological perceptions. Finally, P-OVF exhibits a moderating effect. Specifically, favorable P-OVF is associated with a weak positive effect of leader BLM on teachers' relative deprivation and concurrently moderates the mediating effect of relative deprivation. Furthermore, favorable P-OVF results in a weaker negative effect of leader BLM on teachers' psychological safety and moderates the mediating effect of psychological safety.

### Theoretical Implications

The theoretical significance of this study is mainly reflected in the following aspects: First, we investigates the influence mechanism of leaders' bottom-line mentality on teachers in the Chinese educational context. Leaders' bottom-line mentality is the research frontier in organizational behavior, especially in the context of globalization that pays attention to the quality of economic development. Keeler and Webster (2018) pointed out in the latest research that future research needs to continue to examine the bottom-line mentality of leaders in different organizational and cultural backgrounds. Therefore, the conclusion of this paper on the bottom-line mentality of leaders enriches the theoretical discussion on the bottom-line mentality of leaders in the world, and responds to the research appeal of scholars (Greenbaum et al., 2012; Mesdaghinia et al., 2019).

Secondly, following the research on leaders' bottom-line mentality, it is found that although the bottom-line has played a certain role in ensuring economic profits, scholars have found that if they only pay attention to corporate profits and personal interests, there will be a series of adverse consequences (Sims and Brinkman, 2002; Greenbaum et al., 2012; Bonner et al., 2017; Mawritz et al., 2017; Mesdaghinia et al., 2019). For example, the leader's bottom-line mentality will be transmitted to the employees through an imitation mechanism, which will make the employees have the same bottom-line mentality, and the employees will transform the leader's bottom-line mentality into socially destructive behavior and immoral behavior based on individual cognition. However, whether the bottom-line mentality of leadership will destroy teachers' innovative behavior has not been well-answered. This study found that the bottom-line mentality of leaders will harm the teachers' innovative behavior by influencing their psychological mechanism. Therefore, the research conclusion of this paper confirms the conclusion drawn by many international scholars that leading BLM will have a series of negative consequences for individuals (Sims and Brinkman, 2002; Greenbaum et al., 2012; Bonner et al., 2017; Mawritz et al., 2017; Mesdaghinia et al., 2019), and at the same time enriches the current theoretical research on leadership bottom-line mentality to a certain extent.

Third, relative deprivation and psychological safety played partial mediating roles between leaders' bottom-line mentality and teachers' innovative behavior. First of all, the results of this study clarify the specific impact mechanism of leaders' bottom-line mentality on individuals' innovative behavior, and at the same time expand the theoretical perspective of individuals' pre-factors of innovation. Although previous studies have confirmed the influence of leaders' bottom-line mentality on individuals' behavior, they are mainly based on the perspective of leadership identity and moral identity (Greenbaum et al., 2012; Bonner et al., 2017; Mesdaghinia et al., 2019), and rarely analyze this mechanism from the psychological perspective. Therefore, based on the theory of social exchange, this paper analyzes the influence of leadership bottom-line mentality on teachers' innovative behavior from the psychological level, which is a breakthrough for previous research and provides a new perspective for future related research. Secondly, the fact that teachers' psychological safety and relative deprivation will have a negative impact on individual innovation has been widely confirmed in previous studies (Zigarmi et al., 2009; Hood et al., 2016). Therefore, the research conclusions based on Chinese educational management scenarios once again prove the scholars' researches. It is worth noting that although scholars have revealed some pre-factors that have an impact on individuals' psychological security and relative deprivation from the leadership level, they have not considered the bottom-line mentality of leaders in the research scope. On the basis of the limitations of previous studies, this paper explores a new front factor that affects individuals' psychological security and relative deprivation. Therefore, the conclusion of this paper enriches the theoretical research on individuals' psychological safety and relative deprivation.

Finally, the person-organization values fit has a moderating effect. In previous studies, when the bottom-line mentality of leaders influenced the subordinates, most scholars took moral identity (Mesdaghinia et al., 2019), self-evaluation, and sense of responsibility (Greenbaum et al., 2012) as moderating variables. Differently, we take teachers' person-organization values fit as a moderating variable to explore the boundary conditions of leadership bottom-line mentality, which is a breakthrough. Based on the research conclusion, this paper clarifies the boundary conditions of the effect of leadership bottom-line mentality on individuals' relative deprivation and psychological safety, which enriches the theoretical research on leadership bottom-line mentality (boundary conditions) and expands the literature on the matching of individuals' personal-organizational values fit.

### Managerial Implications

The main purpose of this research is, by referencing the research conclusions, to provide recommendations related to routine education management practices for education and training institutions. Based on the present research conclusions, this study proposes corresponding strategies aimed at sustaining teacher innovation and long-term school education development.

The present study and relevant studies have demonstrated that leader BLM exerts a negative effect on psychological and behavioral aspects of individuals (Greenbaum et al., 2012; Bonner et al., 2017). Therefore, education and training institutions should acknowledge that leaders are role models to teachers in terms of their behavior and attitude and should thus emphasize the importance of leaders being qualified. This study proposes the following approaches to ensuring education and training institutions' leaders are qualified. First, during the education and training institutions' leader recruitment process, the characteristics, qualities, and macro and strategic thinking of candidates should be meticulously examined to prevent the selection of a candidate with potentially high BLM. Second, theoretical knowledge is critical to school education management practices; therefore, education and training institutions should offer training courses on education and training institutions management and thinking skills specifically for leaders so that these leaders can acquire cutting-edge theoretical knowledge, be made aware of the negative influences of BLM on teachers even students, and implement favorable education and training institutions management practices. Third, although leaders with BLM are likely to maximize some education and training institutions' profits, abnormal bottom-line behaviors often lead to negative results. A responsible management approach in education and training institutions is to encourage bottom-line behaviors only at the appropriate time to ensure teachers' well-being while meeting organization expectations regarding the bottom-line. More importantly, it must be acknowledged that how to improve education quality and train talents, and other constructive targets are real leaders affairs of the education and training institutions in daily management.

We find that relative deprivation and psychological safety partly mediate the relationship between leader BLM and teachers' innovative behavior, which makes us have to put forward some suggestions to effectively avoid the negative impact of leader BLM. First of all, we need to find some effective measures to reduce teachers' sense of relative deprivation and improve their psychological safety, so as to alleviate the transmission of negative effects to innovative behaviors. For example, education and training institutions can increase the welfare of teachers, which has been proved to be an effective way to alleviate unfair perception in some literatures. In addition, these institutions can also practice appropriate human resource management to improve individual psychological safety, such as developmental human resource management, inclusive human resource management and committed human resource management. Secondly, partial mediation shows that we can try other ways to transform the negative effects of leader BLM on individual innovation. For example, leader BLM pays more attention to organizational benefits, so we can take appropriate measures to encourage teachers to form performance-oriented approach to goals, such as advocating collective values, which can promote their innovative behavior under the leadership of BLM.

What's more, education and training institutions must acknowledge the importance of innovative teacher behavior in education and training institutions' education development. Education and training institutions can construct an internal learning platform that connects with external platforms to create a learning atmosphere, encourage the formation of learning groups, and thus elicit innovative teacher behavior. This kind of learning method can stimulate teachers' creativity and improve educational practice and teaching quality. Hands-on education activities are essential to fostering innovative teacher behavior; therefore, education and training institutions can hold regular innovation competitions, teaching contest, and educational quality development activities to engage teachers in innovation practices, train their innovative education thinking, and thereby drive their innovative behavior.

### Limitations and Future Directions

Despite obtaining valuable research results, this study had some limitations and there was room for improvement. First, this study investigated only how leader BLM affects teacher innovation behavior; the effect of leader BLM on organizations and other teacher behaviors was not addressed. Accordingly, future research may examine the causal relationship of leader BLM with other factors such as innovation atmosphere in the organization, teachers' organizational commitment, and job burnout. Additionally, this study explained the influence of leader BLM on innovative teacher behavior from the perspective of psychological safety and relative deprivation, but other mediators may also exist. Therefore, scholars can explore the effect from different perspectives to uncover potential mediators. Finally, this study analyzed the moderating effect of P-OVF; however, whether the causal effect of leader BLM is affected by other situational factors has not been determined. Accordingly, other boundary conditions, such as leaders' identity, could be incorporated.

## Data Availability Statement

The raw data supporting the conclusions of this article will be made available by the authors, without undue reservation.

## Ethics Statement

The studies involving human participants were reviewed and approved by the Academic Committee of Business School of Huaqiao University. The patients/participants provided their written informed consent to participate in this study.

## Author Contributions

This study was a joint work of the five authors. LL, WW, and YW contributed to the ideas of educational research, collection of data, and empirical analysis. LL, JL, WW, and QF contributed to the data analysis, design of research methods, and tables. LL, WW, JL, and YW participated in developing a research design, writing, and interpreting the analysis. All authors participated in reading and approval of the final manuscript and have read and agreed to the published version of the manuscript.

## Conflict of Interest

The authors declare that the research was conducted in the absence of any commercial or financial relationships that could be construed as a potential conflict of interest.
